# Longitudinal trends in Gamma Knife radiosurgery utilization across multiple technological generations: A Single‐Institution Observational Study

**DOI:** 10.1002/acm2.70808

**Published:** 2026-10-13

**Authors:** Daniel El Basha, Eric Filiput, Austen Curcuru, Sven Ferguson, Sreekrishna Murty Goddu, Nels Knutson, Hugh Lee, Timothy Mitchell, Seungjong Oh, Joshua L. Dowling, Jiayi Huang, Taeho Kim

**Affiliations:** ^1^ Department of Radiation Oncology Washington University in St. Louis School of Medicine St. Louis Missouri USA; ^2^ Department of Radiation Oncology Barnes Jewish Hospital St. Louis Missouri USA; ^3^ Department of Neurosurgery Washington University in St. Louis School of Medicine St. Louis Missouri USA

**Keywords:** Gamma Knife radiosurgery, intracranial radiosurgery, stereotactic radiosurgery (SRS)

## Abstract

**Background:**

Gamma Knife radiosurgery (GKRS) has been a foundational modality for the treatment of intracranial diseases for several decades, with successive platform generations introducing substantial technological advancements. Improvements in source configuration, patient positioning systems, imaging guidance, and immobilization techniques have expanded clinical capabilities. Despite these ongoing innovations, few long‐term institutional analyses have evaluated whether GKRS utilization has remained stable, increased, or shifted with changes in disease patterns and retreatment needs over time.

**Purpose:**

Gamma Knife radiosurgery (GKRS) has undergone multiple technological evolutions over the past two decades. This study provides a retrospective analysis of longitudinal utilization at a single institution, examining trends in case volume, disease indications, retreatment frequency, and platform era.

**Methods:**

A retrospective analysis of Gamma Knife treatments delivered at one institution between 1998 and 2025 was performed. Annual counts of first, repeat, and total GKRS treatments were analyzed using negative binomial regression with a log link and Newey–West standard errors; platform‐adjusted models accounted for the sequential relationship between calendar time and platform era. Segmented negative binomial regression of monthly counts evaluated slope changes at platform transitions.

**Results:**

Across the study period, 6,201 unique patients received 7,267 delivered treatment courses. Median annual treatment volume was 276 procedures (range, 71–362); median age at first treatment was 60 years (IQR, 50–69), and brain metastasis was the primary indication in 2,991 patients (48.2%). Total volume increased in unadjusted modeling (count ratio [CR], 1.03; *p <* 0.001), and repeat treatments showed the strongest platform‐adjusted growth (CR 1.14; *p <* 0.001). Metastatic disease was the dominant adjusted disease‐specific trend (CR 1.08; *p =* 0.01). Repeat metastatic treatments increased more than overall repeat treatments (CR 1.15; *p <* 0.001). Segmented regression showed significant growth during the baseline Model B era (annualized CR 1.16; *p <* 0.001) followed by significant attenuation at the Model C transition (annualized CR 0.90; *p =* 0.01). The primary segmented model showed no significant incremental slope change at Perfexion or Icon; sensitivity analyses indicated that the estimated Icon‐era association was model‐dependent.

**Conclusions:**

Long‐term Gamma Knife utilization demonstrated sustained institutional growth, with the clearest adjusted temporal trends in metastatic disease and repeat treatment. Because calendar time and sequential platform adoption were collinear, these observational findings describe evolving utilization and practice patterns but do not establish that platform changes caused the observed trends.

## 1 INTRODUCTION

Leksell Gamma Knife radiosurgery (GKRS; Elekta AB, Stockholm, Sweden) has been a cornerstone modality for the management of intracranial pathologies for over half a century. By utilizing multiple cobalt‐60 sources converging at a single isocenter, Gamma Knife achieves highly conformal dose distributions with steep dose gradients, enabling effective tumor control while sparing adjacent normal tissues.^1^, ^2^, ^3^ These characteristics have established GKRS as a standard treatment option for a broad range of intracranial diseases, including brain metastases, meningiomas, vestibular schwannomas, arteriovenous malformations, pituitary adenomas, and functional disorders such as trigeminal neuralgia.^4^, ^5^, ^6^, ^7^, ^8^

Over time, the Gamma Knife platform has undergone multiple technological advancements. Early systems such as Model B and Model C established the foundation of frame‐based radiosurgery, with Model C introducing automated positioning capabilities that improved treatment efficiency.^9^ Subsequent platforms, including Perfexion, redesigned source geometry and collimation systems to expand anatomical reach and streamline workflow.^10^, ^11^, ^12^ More recently, the Icon system introduced cone‐beam CT imaging, motion management, and mask‐based immobilization, enabling frameless and fractionated treatment approaches.^13^, ^14^, ^15^, ^16^ Continued platform evolution, including upgrade pathways such as Esprit, reflects ongoing efforts to enhance precision, flexibility, and integration within modern clinical workflows.^17^, ^18^ The Esprit is not currently used at our institution.

Despite these technological advancements, an important question remains: how has Gamma Knife utilization evolved over time within a consistent clinical environment? Previous studies have demonstrated excellent local control and low toxicity rates across disease sites; however, most reports focus on specific indications or limited time intervals.^19^, ^20^, ^21^, ^22^ While newer platforms introduce capabilities that may influence treatment delivery, their adoption occurs sequentially alongside broader changes in clinical practice. As a result, differences observed across technological generations may reflect both platform‐related changes and underlying temporal trends in radio‐surgical utilization.

In parallel, improvements in systemic therapies have increased patient survival, particularly for metastatic disease, resulting in a growing population at continuing risk for intracranial progression. In selected settings, stereotactic radiosurgery has also reduced reliance on upfront whole‐brain radiotherapy while preserving focal salvage options for subsequent intracranial disease.^23^, ^24^, ^25^ Repeat stereotactic radiosurgery has emerged as a viable salvage strategy, with multiple studies demonstrating acceptable safety profiles and durable tumor control following repeat Gamma Knife treatments.^26^, ^27^, ^28^, ^29^ The ability to deliver subsequent radiosurgery events is therefore relevant to long‐term program planning, although repeat utilization alone is not a measure of safety or efficacy.

A single‐institution Gamma Knife program with continuous operation across multiple technological generations provides a unique opportunity to examine long‐term patterns in radio‐surgical utilization within a consistent clinical environment. Such datasets are particularly valuable for understanding how practice evolves over time, independent of cross‐institutional variability.

Accordingly, the present study was designed as a retrospective observational analysis of longitudinal Gamma Knife utilization. The objectives were to characterize trends in annual treatment volume, evaluate patterns in first and repeat treatments, assess disease‐specific utilization, and explore the relationship between platform era and calendar time. Rather than assessing clinical effectiveness, this work aims to describe how radio‐surgical practice has evolved over time within a single institution.

## 2 METHODS

### 2.1 Study design and data source

A retrospective analysis of Gamma Knife radiosurgery treatment courses delivered between 1998 and 2025 was performed at a large, single academic medical center within a major midwestern metropolitan region. The analytic dataset contained only intracranial treatment courses that were delivered; patients who were evaluated, simulated, or planned but did not receive treatment were not included. Data were de‐identified prior to analysis, and this study was conducted in accordance with institutional policies governing retrospective research.

### 2.2 Data collection and classification

Patient‐level treatment data were extracted from institutional Gamma Knife clinical records. Collected variables included patient identifiers (name, date of birth, and sex), date of treatment, treating attending physician, immobilization method (frame‐based or frameless), prescription dose, and clinical diagnosis. Unique patients were identified using matching name and date of birth. Patient‐level demographic characteristics and primary indication were summarized at the first delivered treatment; treatment‐course characteristics were summarized across all delivered courses.

A treatment event was defined as one delivered Gamma Knife treatment course, regardless of whether the prescription comprised one or multiple targets and fractions. A course was classified as repeat treatment only when a patient with a matching name and date of birth had received a prior Gamma Knife course at the institution on an earlier date. Thus, repeat treatment denotes a subsequent delivered institutional GKRS course for the same patient. Because records were not linked at the lesion level, this definition cannot distinguish retreatment of a previously treated lesion from treatment of a new lesion.

Gamma Knife platform (Model B, Model C, Perfexion, or Icon) was not explicitly recorded in the clinical dataset for all historical treatments. Platform assignment was therefore determined from institutional acceptance‐testing and commissioning dates. Each platform replaced its predecessor at installation; only one platform was in clinical use at a time, and no treatment overlap occurred between platform eras. Each delivered treatment course was assigned to the platform in use on its treatment date, from that platform's commissioning date until the next system's commissioning date.

Disease categories were consolidated to align with standard Gamma Knife diagnostic groupings in Leksell GammaPlan®. Disease sites accounting for less than 1% of the annual case volume were grouped and categorized as ‘other’ to improve statistical robustness and reduce noise from rare indications. This category encompasses a heterogeneous grouping of rare or infrequently treated conditions including atypical schwannomas/nerve‐sheath tumors, uncommon skull‐base tumors (e.g., chordoma, chondrosarcoma, esthesioneuroblastoma, paraganglioma), functional disorders (e.g., essential tremors, Parkinson's disease, dystonia, epilepsy), rare intracranial neoplasms (e.g., craniopharyngioma, hemangioblastoma, lymphoma, germ cell tumors, sarcomas, choroid plexus tumors), and unclassifiable isolated lesions.

### 2.3 Statistical analysis

Statistical modeling was performed to characterize the total number of treatments under the Gamma Knife program over time, identify disease groups contributing most strongly to treatment growth, and determine whether total, first‐time, and repeat treatment counts changed significantly across calendar years and platform eras.

Annual total, first‐time, repeat, and disease‐specific counts were analyzed using negative binomial regression models with a log link. To account for potential serial autocorrelation in repeated observations over time, standard errors were estimated using a Newey–West variance estimator. A lag length of 1 was selected based on inspection of autocorrelation and partial autocorrelation plots of model residuals, which showed minimal evidence of higher‐order temporal dependence. We present count ratios (CRs) and model‐estimated annual percent change in count unadjusted and adjusted for machine era. A CR represents the multiplicative factor by which the expected annual treatment count changes per one‐unit increase in the predictor. For example, a CR of 1.09 for repeat treatments indicates a 9% higher expected count per additional year. CRs greater than 1 reflect increasing treatment count over time, CRs less than 1 reflect decreasing counts, and a CR of 1 indicates no temporal change; in this study, CRs are reported with 95% confidence intervals and used to quantify the magnitude of longitudinal trends in total, first‐time, repeat, and disease‐specific Gamma Knife utilization.

Segmented regression analyses were conducted to evaluate treatment volume across machine eras following a similar model setup. Because platform adoption occurred sequentially over time, models included level (intercept) and slope changes at machine era breakpoints to assess changes in both baseline level and temporal trends at platform transition points. To improve precision in detecting platform transition points, treatment counts were aggregated at the monthly level for segmented regression analyses. The primary segmented model included breakpoints at Model C, Perfexion, and Icon; simplified two‐segment models comparing Model B with all post–Model B eras and the pooled pre‐Icon period with the Icon era were evaluated as sensitivity analyses. Disease‐specific annual counts were analyzed using analogous disease‐specific segmented regression models.

Missing values affected less than 1% of available data and were handled by complete‐case analysis using listwise deletion. Only delivered treatment courses were eligible for analysis. All tests were two‐sided, and *p <* 0.05 or 95% confidence intervals excluding unity were considered statistically significant. Analyzes were performed in Python version 3.13 using statsmodels version 0.14.6.

## 3 RESULTS

### 3.1 Observed trends in treatment utilization

A total of 6,201 unique patients received 7,267 delivered Gamma Knife treatment courses. At first treatment, median patient age was 60 years (IQR, 50–69), and 3,581 patients (57.7%) were female and 2,620 (42.3%) were male. Brain metastasis was the most common primary indication (2,991 patients [48.2%]), followed by trigeminal neuralgia (851 [13.7%]) and meningioma (691 [11.1%]). At the treatment‐course level, median age was 61 years (IQR, 51–69); 6,201 courses (85.3%) were primary and 1,066 (14.7%) were repeat courses. Rigid‐frame immobilization was used for 7,028 courses (96.7%) and mask‐based immobilization for 239 (3.3%); 7,179 courses (98.8%) were single‐fraction and 88 (1.2%) were fractionated (Table 1). Median annual volume was 276 procedures (range, 71–362), with 1998 representing a partial operational year. Annual treatment volume increased across the study period, with the strongest adjusted temporal trend observed for repeat treatments (Figure 1). In unadjusted negative binomial models, total treatment volume increased over time with CR 1.03 (95% CI 1.02–1.04; *p <* 0.001), first‐time treatment volume increased with CR 1.02 (95% CI 1.01–1.03; *p =* 0.001), and repeat treatment volume increased with CR 1.09 (95% CI 1.06–1.12; *p <* 0.001). In platform‐adjusted models, repeat treatments increased with CR 1.14 (95% CI 1.07–1.21; *p <* 0.001), while total treatments and first‐time treatments showed non‐significant positive trends with CR 1.06 (95% CI 1.00–1.12; *p =* 0.05) and CR 1.04 (95% CI 0.99–1.10; *p =* 0.15), respectively. The unadjusted models showed concordant positive temporal trends across all three outcomes, with repeat treatments remaining the largest effect (Table 2).

**TABLE 1:** Patient‐level demographic characteristics and procedure‐level operational and treatment‐planning characteristics for 6,201 unique patients who received 7,267 delivered Gamma Knife radiosurgery treatment courses from 1998 through 2025. Panel A summarizes characteristics at first treatment and course‐level operational parameters; Panel B summarizes course‐level treatment‐planning parameters by diagnostic group.

Panel A. Patient demographics and operational characteristics.		
Characteristic	Data level	Summary statistic
Patient demographics		
Total unique patients	Patient	*N =* 6,201
Age at first treatment, median [IQR], years	Patient	60 [50–69]
Sex, n (%)		
Female	Patient	3,581 (57.7%)
Male	Patient	2,620 (42.3%)
Primary indication at first treatment, n (%)		
Brain metastasis	Patient	2,991 (48.2%)
Trigeminal neuralgia	Patient	851 (13.7%)
Meningioma	Patient	691 (11.1%)
Schwannoma	Patient	575 (9.3%)
Vascular malformation	Patient	473 (7.6%)
Pituitary adenoma	Patient	323 (5.2%)
Glioma	Patient	157 (2.5%)
Other	Patient	140 (2.3%)
Operational parameters		
Total delivered treatment courses	Procedure	*N =* 7,267
Annual procedure volume, median [minimum–maximum]^*^	Procedure	276 [71–362] procedures/year
Annualized mean rate^†^	Procedure	263.8 procedures/year
Monthly mean rate^†^	Procedure	22.0 procedures/month
Age at treatment, median [IQR], years	Procedure	61 [51–69]
Treatment sequence, n (%)		
Primary treatment	Procedure	6,201 (85.3%)
Repeat treatment	Procedure	1,066 (14.7%)
Gamma Knife unit model, n (%)		
Perfexion	Procedure	2,671 (36.8%)
Icon	Procedure	2,579 (35.5%)
Model C	Procedure	1,304 (17.9%)
Model B	Procedure	713 (9.8%)
Immobilization technique, n (%)		
Rigid frame	Procedure	7,028 (96.7%)
Mask‐based	Procedure	239 (3.3%)
Fractionation protocol, n (%)		
Single‐fraction	Procedure	7,179 (98.8%)
Fractionated	Procedure	88 (1.2%)

Panel B. Treatment‐planning parameters stratified by diagnostic group.					
Diagnostic group	n (%)	Marginal dose (Gy), median [IQR] (range)	Isodose line (%), median [IQR] (range)	Lesions, median [IQR] (range)	Fractions, median [IQR] (range)
Brain metastasis	3,806 (52.4%)	20 [18–20] (5–80)	50 [50–50] (5–90)	2 [1–3] (1–19)	1 [1–1] (1–5)
Trigeminal neuralgia	962 (13.2%)	40 [40–40] (12–82)	50 [50–50] (40–100)	1 [1–1] (1–2)	1 [1–1] (1–1)
Meningioma	755 (10.4%)	15 [14–16] (5–40)	50 [50–50] (5–80)	1 [1–1] (1–9)	1 [1–1] (1–5)
Schwannoma	582 (8.0%)	12 [12–13] (5–25)	50 [50–50] (25–80)	1 [1–1] (1–2)	1 [1–1] (1–5)
Vascular malformation	519 (7.1%)	18 [16–18] (10–24)	50 [50–50] (20–60)	1 [1–1] (1–6)	1 [1–1] (1–1)
Pituitary adenoma	329 (4.5%)	18 [16–20] (5–25)	50 [50–50] (20–70)	1 [1–1] (1–2)	1 [1–1] (1–5)
Glioma	165 (2.3%)	18 [16–20] (5–24)	50 [50–50] (25–60)	1 [1–1] (1–4)	1 [1–1] (1–5)
Other	149 (2.1%)	17 [15–20] (8–80)	50 [50–50] (45–80)	1 [1–1] (1–14)	1 [1–1] (1–5)

Abbreviations: Gy, gray; IQR, interquartile range (25th–75th percentiles); n, subgroup count; N, total patient or treatment‐course count as indicated.

Patient‐level characteristics were summarized once per unique patient at the first delivered treatment. Procedure‐level characteristics were summarized across all delivered treatment courses; patients receiving repeat treatment could contribute more than one course.

*Annual procedure volume is reported across all operational calendar years (1998–2025; 28 years). The minimum occurred during the partial launch year (1998; *n =* 71), and the maximum occurred in 2025 (*n =* 362). Restricting the summary to full calendar years (1999–2025; 27 years) yields a median of 278 procedures/year (range, 141–362).

†Annualized and monthly mean procedure rates were calculated across the 27.55‐year service interval from June 1998 through December 2025.

Continuous variables are reported as median [IQR] or median [IQR] (range), as specified; categorical variables are reported as n (%). Panel B is summarized at the delivered‐treatment‐course level.

**FIGURE 1:**
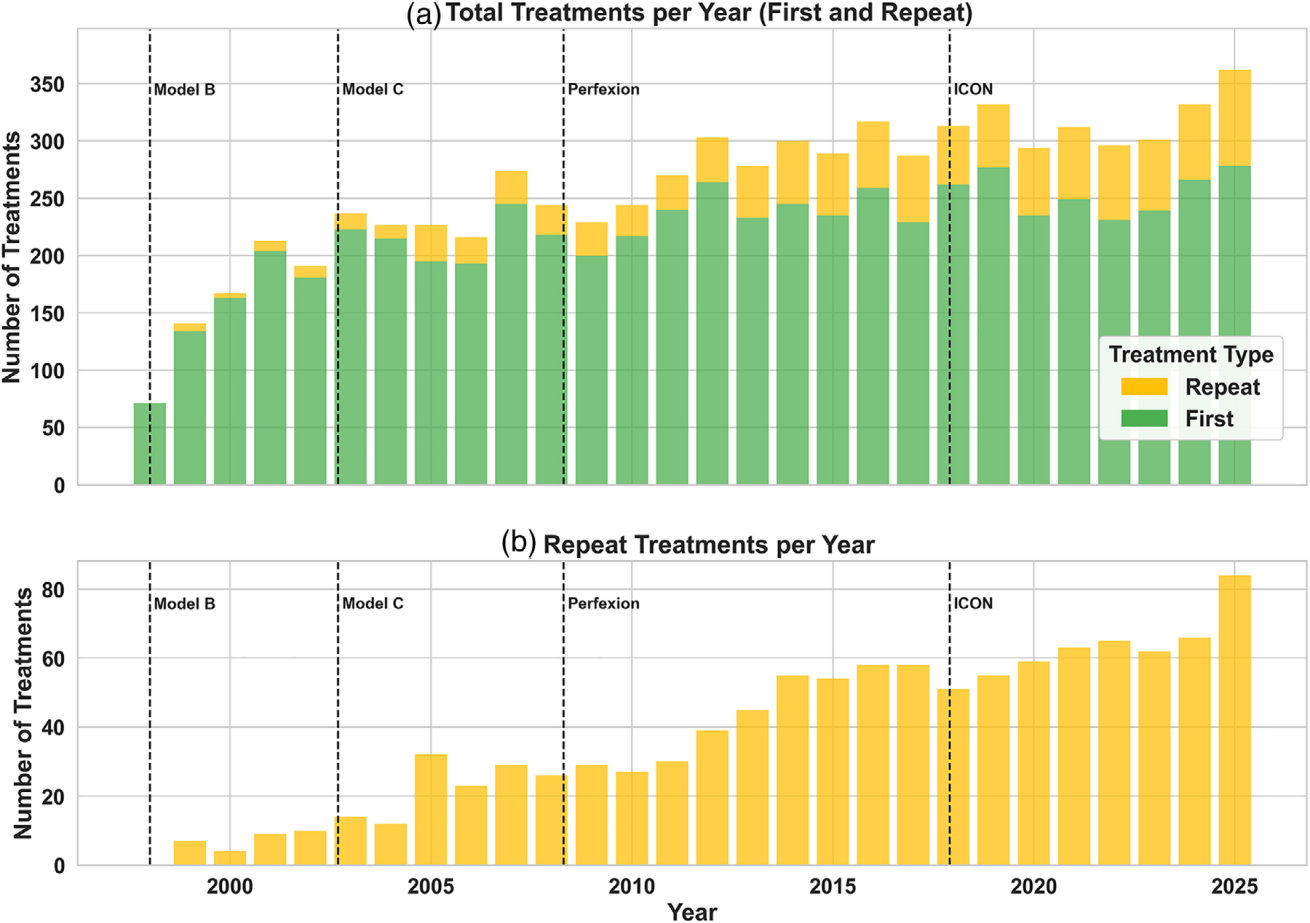
Annual number of delivered Gamma Knife treatment courses from 1998 to 2025, categorized by annual first‐time treatment courses and repeat treatment courses (a) and repeat treatment courses only (b).

**TABLE 2:** Annual negative binomial regression estimates for total, first‐time, repeat, and metastatic delivered treatment‐course counts.

Treatment category	Unadjusted CR	95% CI	p value	Adjusted CR^*^	95% CI	p value
Total treatments	1.03	1.02, 1.04	<0.001	1.06	0.999, 1.12	0.053
First‐time treatments	1.02	1.01, 1.03	0.001	1.04	0.99, 1.10	0.148
Repeat treatments	1.09	1.06, 1.12	<0.001	1.14	1.07, 1.21	<0.001
Total treatments (metastasis only)	1.05	1.04, 1.07	<0.001	1.08	1.02, 1.14	0.010
First‐time treatments (metastasis only)	1.04	1.03, 1.06	<0.001	1.06	1.00, 1.12	0.094
Repeat treatments (metastasis only)	1.09	1.07, 1.12	<0.001	1.15	1.07, 1.22	<0.001

CR denotes the multiplicative change in expected annual count per calendar year. * Adjusted models include Gamma Knife platform era.

### 3.2 Disease‐specific trends

Disease‐specific utilization showed the strongest and most consistent growth for metastatic disease (Figure 2). In the unadjusted disease‐specific negative binomial model, metastatic disease increased with CR 1.05 (95% CI 1.04–1.07; *p <* 0.001), and in the platform‐adjusted model, metastatic disease increased with CR 1.08 (95% CI 1.02–1.14; *p =* 0.01), where the CR represents the multiplicative change in expected annual treatment count per one‐year increase at the center of the study period. Other statistically significant unadjusted trends included increases for meningioma with CR 1.03 (95% CI 1.01–1.04; *p <* 0.001) and schwannoma with CR 1.02 (95% CI 1.00–1.05; *p =* 0.03), and decreases for glioma with CR 0.91 (95% CI 0.89–0.93; *p <* 0.001) and pituitary adenoma with CR 0.97 (95% CI 0.95–0.99; *p =* 0.01). After platform adjustment, schwannoma also increased significantly with CR 1.06 (95% CI 1.00–1.12; *p =* 0.003), while meningioma, trigeminal neuralgia, other diagnoses, vascular indications, pituitary adenoma, and glioma did not reach statistical significance. The unadjusted and adjusted models identify metastatic disease as the dominant disease‐specific growth trend, with additional model‐dependent evidence of schwannoma growth (Table 3).

**FIGURE 2:**
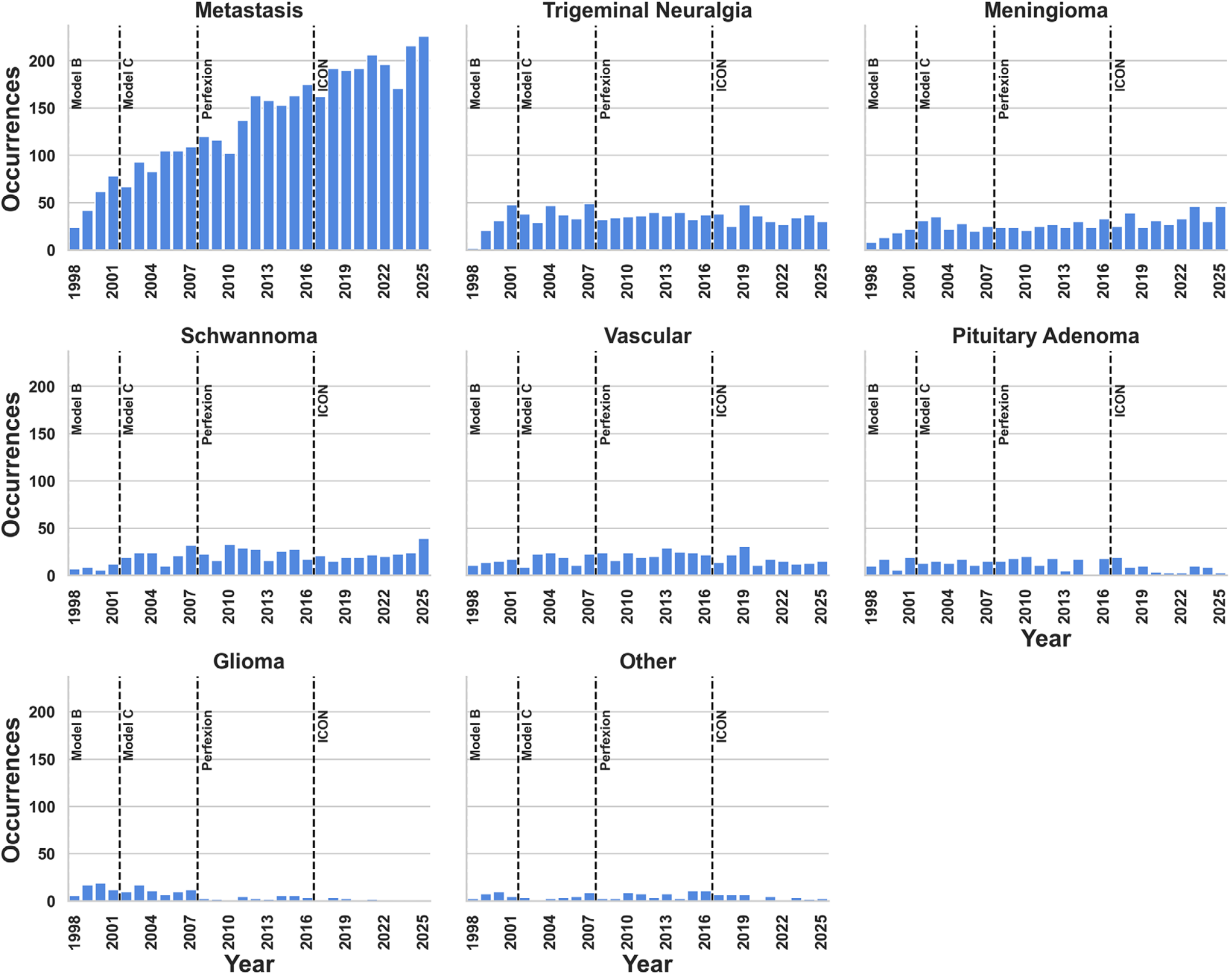
Annual frequency of delivered intracranial Gamma Knife treatment courses at our center from 1998 to 2025. Conditions include metastasis, trigeminal neuralgia, meningioma, schwannoma, vascular lesions, pituitary adenoma, glioma, and other diagnoses.

**TABLE 3:** Annual negative binomial regression estimates by diagnostic category.

Diagnostic category	Unadjusted CR	95% CI	p value	Adjusted CR^*^	95% CI	p value
Glioma	0.91	0.89, 0.93	<0.001	0.96	0.87, 1.06	0.374
Meningioma	1.03	1.01, 1.04	<0.001	1.05	0.99, 1.12	0.093
Metastasis	1.05	1.04, 1.07	<0.001	1.08	1.02, 1.14	0.012
Other	0.99	0.96, 1.02	0.527	1.05	0.96, 1.16	0.276
Pituitary adenoma	0.97	0.95, 0.99	0.007	0.98	0.92, 1.03	0.419
Schwannoma	1.02	1.00, 1.05	0.029	1.06	1.00, 1.12	0.003
Trigeminal neuralgia	1.01	0.99, 1.03	0.624	1.05	0.99, 1.11	0.126
Vascular	1.00	0.99, 1.02	0.786	1.00	0.93, 1.05	0.802

CR denotes the multiplicative change in expected annual count per calendar year. * Adjusted models include Gamma Knife platform era.

### 3.3 Repeat treatments

Repeat treatments represented a distinct and clinically meaningful temporal trend within the longitudinal utilization pattern (Figure 1). In the primary platform‐adjusted negative binomial model, repeat treatments increased with CR 1.14 (95% CI 1.07–1.21; *p <* 0.001). Among metastatic disease treatments, repeat treatments increased with CR 1.15 (95% CI 1.07–1.22; *p <* 0.001), exceeding the overall repeat‐treatment growth estimate and identifying metastatic retreatment as the strongest repeat‐treatment contributor (Figure 3). Total metastatic treatments increased with CR 1.08 (95% CI 1.02–1.14; *p =* 0.01), whereas first‐time metastatic treatments did not reach statistical significance with CR 1.06 (95% CI 1.00–1.12; *p =* 0.09). In the unadjusted negative binomial models, repeat treatments increased with CR 1.09 (95% CI 1.06–1.12; p < 0.001), and repeat metastatic treatments increased with CR 1.09 (95% CI 1.07–1.12; *p <* 0.001) (Table 2).

**FIGURE 3:**
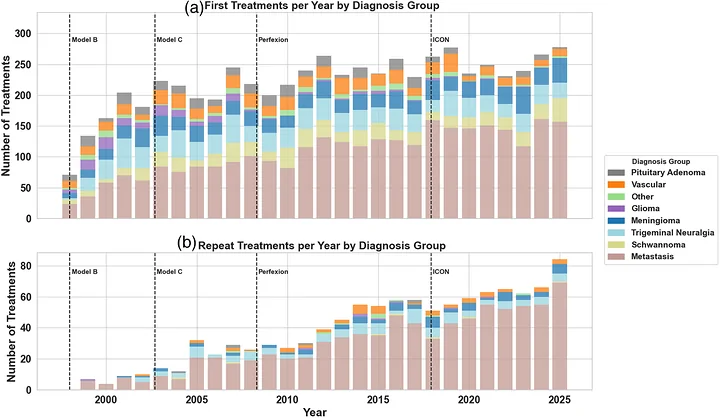
Temporal trends in delivered Gamma Knife treatment courses by diagnostic group for first and repeat treatments. Stacked bar plots show the annual number of delivered treatment courses stratified by diagnostic group for (a) first‐time Gamma Knife radiosurgery treatments (top panel) and (b) repeat Gamma Knife treatments (bottom panel). Vertical dashed lines denote transitions between Gamma Knife platform eras at the institution (Model B, Model C, Perfexion, and Icon).

### 3.4 Platform‐specific utilization

Utilization trajectories changed across platform eras, with the clearest structural change occurring after the earliest Model B period. In the primary monthly segmented model, the baseline Model B time trend increased with an annualized CR of 1.156 (95% CI, 1.075–1.256; *p <* 0.001). The transition to Model C was associated with significant attenuation of this slope, with an annualized slope‐change CR of 0.895 (95% CI, 0.825–0.976; *p =* 0.012). The incremental slope‐change terms at the Perfexion and Icon transitions were not statistically significant, with annualized slope‐change CRs of 0.996 (95% CI, 0.965–1.037; *p =* 0.866) and 0.986 (95% CI, 0.965–1.012; *p =* 0.216), respectively. These slope‐change ratios quantify changes relative to the preceding trajectory and should not be interpreted as absolute within‐era trends. The primary model therefore indicates rapid initial Model B growth followed by significant attenuation at the Model C transition, without evidence of additional sustained slope changes at the later Perfexion or Icon transitions.

Observed annual treatment totals and aggregated monthly model predictions showed descriptive agreement across the study interval (Figure 4). In a simplified sensitivity analysis comparing Model B with all post‐Model B eras, the post‐Model B slope was significantly attenuated relative to the baseline Model B slope (annualized slope‐change CR, 0.882; 95% CI, 0.815–0.953; *p =* 0.002). In a separate sensitivity analysis comparing the pooled pre‐Icon period with the Icon era, the Icon‐associated slope was also modestly attenuated (annualized slope‐change CR, 0.981; 95% CI, 0.965–0.999; *p =* 0.034). The simplified Icon model also estimated an immediate level decrease at the Icon transition (level CR, 0.871; 95% CI, approximately 0.789–0.961; *p =* 0.006). Because the simplified analysis pooled the heterogeneous Model B, Model C, and Perfexion eras into a single pre‐Icon trajectory, whereas the primary model accounted for the earlier transitions separately, the Icon‐era finding was considered model‐dependent. Neither analysis permits attribution of the observed utilization pattern specifically to the Icon platform because platform era and calendar time were sequentially collinear (Table 4).

**FIGURE 4:**
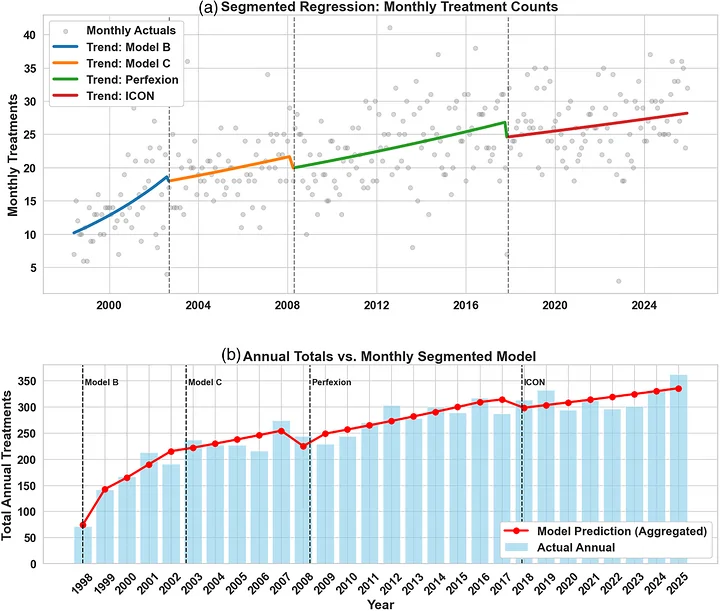
(a) Piecewise segmented regression analysis of monthly delivered Gamma Knife treatment‐course volumes (1998–2025). The plot illustrates raw monthly counts (gray dots) overlaid with regression‐predicted trends for each technology era. Vertical dashed lines denote the exact installation dates of Model C, Perfexion, and Icon, highlighting the distinct shifts in growth rates between machine generations. (b) Comparison of actual annual delivered treatment totals (bars) versus the aggregated monthly segmented regression model (line). Vertical dashed lines indicate mid‐year transition points for new platform installations.

**TABLE 4:** Monthly and annualized count ratios from segmented regression of delivered treatment volume by Gamma Knife platform era.

Treatment era	Monthly CR	95% CI	p value	Annual CR	95% CI	p value
Model B	1.012	1.01, 1.02	<0.001	1.156	1.075, 1.256	<0.001
Model C	0.991	0.98, 1	0.012	0.895	0.825, 0.976	0.012
Perfexion	1.000	1, 1	0.866	0.996	0.965, 1.037	0.866
ICON	0.999	1, 1	0.216	0.986	0.965, 1.012	0.216
Model B^*^	1.012	1.01, 1.02	<0.001	1.156	1.075, 1.256	<0.001
Post Model B^**^	0.990	0.98, 1	0.002	0.882	0.815, 0.953	0.002
Pre ICON^**^	1.003	1.002, 1.004	<0.001	1.037	1.024, 1.049	<0.001
ICON^*^	0.998	0.997, 1	0.034	0.981	0.965, 0.999	0.034

*Single platform era evaluated against all other eras.

**Grouped eras; for example, post–Model B includes Model C, Perfexion, and Icon.

## 4 DISCUSSION

Longitudinal Gamma Knife utilization demonstrated a sustained temporal pattern in which repeat treatments showed the clearest adjusted growth trend. Although total and first‐time treatment volumes also trended upward, their platform‐adjusted temporal estimates were not statistically significant. Repeat treatment was the most robust adjusted utilization signal, not proof of greater efficacy or a platform effect. This pattern situates repeat radiosurgery as an increasingly prominent part of long‐term Gamma Knife practice within a modality that remains established for malignant, benign, vascular, and functional intracranial indications.^3^, ^4^, ^5^, ^6^, ^7^, ^8^

The most prominent disease‐specific expansion in this cohort was observed for metastasis after adjustment for platform era. That finding is concordant with the broader evolution of intracranial radiosurgery, in which stereotactic radiosurgery has assumed an increasingly central role in the management of brain metastases because of expanding indications, reduced reliance on whole‐brain radiation in selected settings, and improved survival associated with modern systemic therapies.^4^, ^17^, ^18^, ^19^, ^20^, ^21^, ^22^, ^23^ The absence of significant adjusted temporal effects for the remaining disease groups indicates that long‐term utilization growth was not distributed evenly across the diagnostic spectrum. These contextual developments are plausible contributors, but the present dataset does not measure systemic therapy exposure, survival, imaging frequency, or treatment‐selection decisions and therefore cannot determine why metastatic utilization increased. The adjusted association should not be interpreted as a platform effect because platform era and calendar time were sequentially collinear.^21^, ^22^, ^23^ Recent randomized data in patients with 5–20 brain metastases showed that stereotactic radiation, compared with hippocampal‐avoidance whole‐brain radiation, improved patient‐reported symptom severity and interference with daily functioning.^30^ These findings support the expanding contemporary role of focal treatment for multiple brain metastases, but they do not explain this institution's longitudinal utilization pattern.

Repeat Gamma Knife treatments also increased substantially over time and represented the most stable treatment‐volume finding across the analyses. However, the patient‐level definition captures any subsequent institutional GKRS event and cannot distinguish same‐lesion reirradiation or local failure from treatment of a new lesion or regional intracranial failure. The observed increase therefore represents repeat utilization, not evidence about recurrence pattern, lesion‐level efficacy, or safety. This observation is consistent with the growing use of repeat radiosurgery as a salvage or retreatment strategy for recurrent or metachronous intracranial disease, particularly in the setting of prolonged survival and continuing intracranial risk.^21^, ^23^, ^24^, ^25^, ^26^, ^27^ Prior reports have shown that repeat Gamma Knife radiosurgery can be integrated across multiple disease settings with acceptable safety and continued clinical utility, including brain metastases, trigeminal neuralgia, vestibular schwannoma, and other recurrent intracranial pathology.^24^, ^25^, ^26^, ^27^ Within that context, repeat treatment represents an increasingly important capacity consideration for long‐term Gamma Knife practice.

Platform‐era analyses showed a transition from early Model B growth to a more stable post‐Model B trajectory. Platform evolution has introduced automated positioning, sector‐based collimation, broader anatomical reach, and later cone‐beam CT‐guided frameless treatment with motion monitoring ^9–16^. These capabilities can support flexible intracranial workflows, but this utilization dataset does not measure dosimetry, treatment time, throughput, staffing, or patient outcomes and cannot compare clinical or operational performance. The segmented results describe a maturing program in which the most pronounced structural change occurred after the earliest platform era, while later transitions did not show additional significant changes in slope. Other radiosurgery platforms and evolving techniques may influence future referral and treatment patterns, so this institutional Gamma Knife trajectory should not be extrapolated as a forecast of the broader SRS landscape. National data from 2003–2011 documented increasing LINAC‐based SRS use, with adoption patterns varying by facility type and geographic region.^31^ Because the present dataset is limited to one institutional Gamma Knife program and does not contain comparative LINAC utilization, its trajectory should not be interpreted as a proxy for the broader SRS market.

Taken together, these findings characterize long‐term Gamma Knife utilization as a practice‐pattern trajectory shaped most strongly by repeat treatment growth and metastatic disease. Sustained utilization across platform eras is best interpreted as evidence of continued institutional integration of Gamma Knife radiosurgery rather than direct evidence of clinical superiority or treatment efficacy. Program maturation, changing referral networks, physician preferences, imaging, systemic therapy, and availability of competing treatment technologies could all contribute, but none was measured as a causal determinant. For programs planning long‐term SRS services, the observed pattern supports anticipating repeat‐treatment capacity, maintaining adaptable scheduling and immobilization workflows, and investing in longitudinal data systems that can link treatment events to lesions and outcomes. These are programmatic implications rather than evidence of throughput, safety, or comparative effectiveness.^20^, ^26^

### 4.1 Limitations

This analysis is limited by its retrospective, single‐institution design. The study was performed at a large academic medical center within a major Midwestern metropolitan region, but the dataset was limited to the institutional Gamma Knife program and did not include the institution's LINAC‐based SRS program or regional competitor volumes. Program maturation and regional recognition may be plausible contributors to referral growth, but referral sources and institutional reputation were not measured. These factors cannot be separated from longitudinal utilization trends, and the observed trajectory should not be generalized to all institutional or regional SRS care. Nevertheless, continuity within a single Gamma Knife program permits long‐term changes in treatment volume, disease composition, repeat utilization, and delivery technique to be characterized without inter‐institutional variability.

The analysis evaluates utilization rather than lesion‐level clinical outcomes. The dataset does not include uniform long‐term measures of local control, survival, radiation necrosis, neurologic toxicity, or patient‐reported outcomes across the full study interval. Increasing treatment volume therefore cannot be interpreted as direct evidence of therapeutic efficacy. Despite this, longitudinal utilization remains informative as a practice‐pattern endpoint when considered in the context of established radiosurgery literature across malignant, benign, vascular, and functional disorders.^4^, ^5^, ^6^, ^7^, ^8^

Interpretation of platform‐era effects is constrained by the sequential relationship between calendar year and machine adoption. Each Gamma Knife platform entered clinical use during a distinct time interval, so platform era and time are inherently collinear in this institutional time‐series structure. Segmented regression and platform‐adjusted count models describe temporal patterns in the presence of this structure, but they do not fully separate technological effects from secular changes in referral patterns, disease management, or institutional growth. Nevertheless, the segmented results identify where treatment‐volume trajectories changed most strongly over time and place later platform‐era comparisons within the context of the program's longitudinal maturation.

Model‐specific limitations affect several statistical estimates. Negative binomial regression was selected to account for overdispersion in count outcomes; however, estimates from lower‐volume disease groups were less precise and more sensitive to model specification. In addition, the sequential relationship between calendar time and platform era limits separation of platform‐associated effects from broader secular trends. These limitations do not affect the descriptive treatment counts but require cautious interpretation of adjusted platform‐era estimates.

The study is limited in its ability to make direct statements about workflow efficiency or throughput. Treatment records showed that rigid‐frame immobilization was used for 7,028 of 7,267 delivered courses (96.7%) and mask‐based immobilization for 239 (3.3%). However, no immobilization‐specific temporal model or causal decomposition was performed. Because mask availability coincided with later platform and calendar eras, these data cannot isolate whether frame‐based versus frameless treatment altered annual case volume. The absence of a significant incremental Icon slope change in the primary segmented model does not establish the absence of an immobilization effect, particularly because the simplified pre‐Icon versus Icon analysis produced a model‐dependent attenuation and mask availability coincided with the Icon and later calendar eras. The dataset also does not include treatment duration, setup time, room utilization, staffing, or scheduling efficiency. This design nonetheless shows that mask‐based and fractionated treatments were incorporated into an active Gamma Knife program, which remains informative considering published reports supporting the technical accuracy and feasibility of frameless Gamma Knife radiosurgery.^13^, ^14^, ^15^, ^16^

Disease‐specific analyses are influenced by changing indications, referral thresholds, imaging practices, competing therapies, and patient selection over time. The clinical meaning of annual count changes differs across diseases, and lower‐volume diagnostic groups are more sensitive to model specification and limited statistical precision. These features limit the strength of inference for diagnoses other than metastasis. Nevertheless, metastatic disease and repeat metastatic treatment showed the clearest adjusted growth trends, supporting the conclusion that the strongest longitudinal utilization pattern was concentrated in metastatic retreatment.

## 5 CONCLUSIONS

This study provides a longitudinal observational analysis of Gamma Knife radiosurgery utilization over nearly three decades of clinical practice spanning multiple technological generations. Across this interval, annual treatment volume increased descriptively, and repeat‐treatment utilization showed the clearest adjusted temporal growth. Total and first‐time platform‐adjusted estimates did not demonstrate statistically significant temporal changes.

Temporal trends were associated with increasing utilization, but the study cannot determine whether changes in disease management, survival, referral patterns, imaging, or other practice factors caused the observed trajectory. Platform‐era associations should be interpreted within the context of sequential technological adoption and calendar‐time collinearity.

Importantly, the present study was not designed to assess clinical effectiveness, workflow efficiency, or comparative performance between platforms. Rather, these results describe how Gamma Knife utilization has evolved within a single institutional setting over time.

These findings highlight the value of long‐term institutional datasets for characterizing radiosurgical practice and planning repeat‐treatment capacity. Future studies incorporating lesion‐level linkage, patient outcomes, referral and competing‐modality data, and workflow metrics will be necessary to explain the clinical and operational implications of these trends.

## AUTHOR CONTRIBUTIONS

**Daniel El Basha**—Data Analysis and manuscript writing, **Taeho Kim**—Study design, manuscript review, Data Analysis, manuscript review, **Data Collection**—Eric Filiput, Austen Curcuru, Sven Ferguson, Sreekrishna Murty Goddu, Nels Knutson, Hugh Lee, Timothy Mitchell, Seungjong Oh, Joshua L. Dowling, Jiayi Huang

## CONFLICT OF INTEREST STATEMENT

The authors declare no conflict of interest.

## ETHICAL APPROVAL

This research was conducted in accordance with the principles embodied in the Declaration of Helsinki and in accordance with local statutory requirements. The retrospective study was approved (IRB# 202307005) by the Human Research Protection Office at the Washington University in St. Louis, MO, USA. The need for written consent was waived owing to the retrospective nature of the study.

## Data Availability

The data that support the findings of this study are not publicly available due to the inclusion of protected health information and institutional restrictions related to patient privacy. Access to manuscript data may be considered upon reasonable request to the corresponding author and with appropriate institutional approvals.
